# Analysis of the uptake and associated factors for virtual crisis care during the pandemic at a 24-h mental health crisis centre in Manitoba, Canada

**DOI:** 10.1186/s12888-022-04166-w

**Published:** 2022-08-04

**Authors:** Tanvi Vakil, Danielle Carignan Svenne, James M. Bolton, Depeng Jiang, Sasha Svenne, Jennifer M. Hensel

**Affiliations:** 1 Rady School of Medicine, University of Manitoba, 750 Bannatyne Ave., Winnipeg, MB Canada; 2 Crisis Response Centre, 817 Bannatyne Ave., Winnipeg, MB Canada; 3 Department of Psychiatry, University of Manitoba, 771 Bannatyne Ave, Winnipeg, MB Canada; 4 Department of Community Health Sciences, University of Manitoba, 750 Bannatyne Ave, Winnipeg, MB Canada; 5 Department of Psychology, University of Winnipeg, 515 Portage Ave., Winnipeg, MB Canada

**Keywords:** COVID-19, Coronavirus, Virtual care, Crisis care, Mental health

## Abstract

**Background:**

The coronavirus pandemic necessitated the rapid transition to virtual care. At a 24-h walk-in mental health Crisis Response Centre (CRC) in Winnipeg, Canada we adapted crisis mental health assessments to be offered virtually while the crisis centre also remained open to in person visits. Little is known about the sustainability of virtual visits in the presence of comparable in person care, and which visits are more likely to be done virtually, particularly in the crisis setting.

**Methods:**

An analysis of visits to the CRC from the first local lockdown on March 19, 2020 through the third local wave with heightened public health restrictions in June 2021. Analysis of Variance was used to compare the proportion of visits occurring virtually (telephone or videoconference) during the first wave of heightened public health restrictions (lockdown 1) and subsequent lockdowns as well as the in-between periods. A binary logistic regression examined visit, sociodemographic and clinical factors associated with receipt of a virtual visit compared to an in person visit over the first year of the pandemic.

**Results:**

Out of 5,357 visits, 993 (18.5%) occurred virtually. There was a significant difference in proportion of virtual visits across the pandemic time periods (*F*(4, 62) = 8.56, *p* < .001). The proportion of visits occurring virtually was highest during lockdown 1 (mean 32.6% by week), with no differences between the other time periods. Receipt of a virtual visit was significantly associated with daytime weekday visits, age, non-male gender, living further away from the CRC, no prior year contact with the CRC, and visits that did not feature suicidal behaviour, substance use, psychosis or cognitive impairment.

**Conclusions:**

A large proportion of virtual care occurring at the outset of the pandemic reflects public anxiety and care avoidance paired with health system rapid transformation. The use of virtual visits reduced over subsequent pandemic periods but was sustained at a meaningful level. Specific visit, sociodemographic and clinical characteristics are more likely to be present in visits occurring virtually compared to those in person. These results can help to inform the future planning and delivery of virtual crisis care.

## Introduction

Coronavirus disease 2019 (COVID-19) has been one of the most consequential events in the twenty-first century, causing radical changes in the structure of our society and health care systems [1, 2]. The pandemic has had a significant impact on the mental health of the general population, especially on people with pre-existing psychiatric and substance use disorders [3, 4]. Social distancing and isolation strategies were implemented worldwide to mitigate and control the spread of the virus. To comply with these efforts, the healthcare system had to adapt and rapidly adopt virtual care, including care delivered by telephone and videoconference [5, 6].

With COVID-19 necessitating widespread adoption of virtual care delivery at a population level, the question is now not if it is feasible, but how and to whom we should deliver this care. While there has been some emerging work into understanding the delivery of care via videoconferencing to home-based settings for routine outpatient mental health care [7–10], there is a gap when it comes to the provision of exclusive use of virtual care in the context of mental health crises. Although telephone-based crisis care, such as the suicide hot-line, is commonly used to provide urgent support to individuals in their homes, this support is usually limited to evaluating the imminent risk and providing immediate interventions to reduce this risk [11]. The usual breadth of comprehensive crisis assessment and support is provided through in-person contact occurring with crisis outreach teams, in emergency departments and urgent care settings, and through specialized clinics [12]. Given the high risk inherent in these presentations, the delivery of virtual care requires special considerations. Crisis presentations can certainly be assessed remotely; emergency telemental health programs have been effective at reducing gaps in mental health human resources in both rural and urban settings across several jurisdictions [13–15]. These assessments, however, are conducted with individuals at another health care facility and supervised by health professionals. The pandemic saw novel adaptations of home-based virtual care delivery in some acute care settings, such as a virtual urgent psychiatric triaging and referral service [16] and partial hospitalization services [17]. These examples further demonstrate the feasibility of providing virtual at-home intervention for higher acuity mental health needs, but offer limited insight into the delivery of comprehensive virtual crisis assessment and how to select which patients receive virtual versus in person treatment.

To adapt to the pandemic, the Crisis Response Centre (CRC) in Winnipeg, Canada rapidly virtualized the full spectrum of crisis services to keep individuals at home whenever possible [18]. This included conducting comprehensive mental health assessments (MHAs) virtually in a non-scheduled clinical setting for high acuity mental health presentations. The prolonged imposed state of lockdown due to the pandemic presents a unique opportunity to study the uptake of virtual crisis services at the CRC. Since services continued to be concurrently offered in-person, a naturalistic comparison group was available. This study examined the uptake of virtual visits for crisis mental health care during the COVID-19 pandemic and evaluated the characteristics and outcomes of visits occurring virtually compared to those in person.

## Methods

### Setting

The CRC is located in the city of Winnipeg, Canada (population ~ 760,000) and provides 24-h, 7 day a week services for adults age 18 and over experiencing a mental health crisis. It is a centralized facility serving the entire city and provides specialized urgent mental health assessment and treatment without requiring a formal referral. The facility houses both a walk-in service and crisis phone line offering brief risk assessment and support (on average calls last about 10 min), but does not have inpatient beds. Crisis calls needing further assessment are directed to attend in person at the CRC or escalated to emergency response services.

Services are provided with a collaborative team approach in a stepped care model. Mental health clinicians with training in social work, occupational therapy or psychiatric nursing are the first step in care and will conduct a comprehensive MHA over approximately 60–90 min. Specific clinical indicators lead to a subsequent psychiatric assessment after the MHA on the same visit. Individuals can be admitted to inpatient psychiatric units, transferred to a community crisis stabilization unit (CSU), or discharged to outpatient services.

### Virtual adaptations in crisis care

As the COVID-19 pandemic unfolded, the CRC adapted its processes in order to provide the option of virtual crisis assessment services [18]. The walk-in facility remained open to ensure equitable population access, and the crisis line continued to operate in its usual capacity for brief support calls. However, additional pathways were launched to reduce risk of COVID-19 spread and to attend to public anxiety about accessing facility-based services. Signage posted at the front of the building directed users to contact the CRC by phone if they felt able to wait and receive a virtual assessment. Individuals who were triaged as lower mental health acuity or higher COVID risk were redirected to return home and receive a virtual assessment, and those calling in through the mobile crisis line could be referred for a virtual MHA rather than being directed to attend on site as was usual pre-pandemic practice. The decision to offer a virtual MHA was collaborative and based on client preference, acuity of mental health crisis, COVID status, safety to remain in the community, and the number of individuals already receiving services on site. Communication about these expanded options for care was also sent out by email to community partners and referral sources. Practice change was supported by the creation of standard operating procedures, scripts, consent procedures, flexibility for onsite or remote work, email communications, and daily huddles. During the pandemic, a virtual ward was launched to provided intensive (daily) crisis intervention virtually through a team of mental health clinicians, physician assistants and psychiatrists to account for reduced capacity in the physical facilities due to social distancing and bed spacing requirements. The virtual ward was an available disposition following an MHA.

### Data sources

All data were extracted from the CRC’s electronic patient record (EPR) for all visits during the study period. The EPR contains data on all CRC visits, and assessment details are entered by the assessing clinicians. All individuals are identified by a unique CRC file number, and all visits are assigned a unique visit number. We obtained all visits from March 19, 2020 to June 30, 2021 to examine trends in virtual care uptake across key phases of the pandemic. We created a comparison sample of visits during roughly the first year (March 19, 2020 to April 7, 2021) to compare those visits done in person to those done virtually. For the comparison sample, we also obtained visits during the 1-year lookback period (to March 19, 2019) to examine pre-visit contact with the CRC. The EPR forms contain a combination of forced entry and free text fields. Any free text responses were manually classified. In the case of missing data (eg. postal code which was missing for 673 individuals), the team conducted a manual chart review to retrieve the data where possible.

Research ethics approval for the study was obtained from the University of Manitoba Research Ethics Board (HS23878 (H2020:196)).

### Variables

#### Type of visit

All visits were coded as in-person (individual seen onsite at the CRC) or virtual (assessment delivered virtually via telephone or videoconference).

#### Visit characteristics

We coded the day of the week (weekday – Monday through Thursday, weekend – Friday through Sunday), time of day corresponding to typical staff shifts (7am to 3 pm, 3 pm to 11 pm, and 11 pm to 7am) and period of the pandemic when the visit occurred. The pandemic periods aligned with the level of restrictions in place for the Winnipeg area. Times of heightened restrictions are designated as “lockdown” periods where citizens were instructed to limit contact outside of their homes, gatherings were significantly restricted, and non-essential businesses were closed. These dates corresponded to 5 time periods: start of study/lockdown 1 (March 19, 2020 to May 4, 2020), between lockdown 1 and 2 (May 5, 2020 to Sept 27, 2020), lockdown 2 (Sept 28, 2020 to Feb 12, 2021), between lockdown 2 and 3 (Feb 13, 2021 to April 20, 2021) and lockdown 3 (April 21, 2021 to the end of the study period, June 30, 2021) [19].

#### Sociodemographics

Age at time of visit was calculated as the difference in years between visit date and date of birth. Gender was coded as male, female or other. Distance between the individual’s residence and the CRC was calculated as the distance in kilometers (km) from the CRC’s address and the geographic centre of the forward sortation area (FSA) corresponding to the first 3 digits of the individual’s postal code. To determine the geographic centre of the FSA, the Statistics Canada boundary files [20] were downloaded in cartographic form and loaded onto the software qGIS in the Lambert Conformal Conic Projection (EPSG 3347) and the geographical centres were collected using the feature Vector/Geometry Tools/Centroids. The address for the CRC was entered using the geocoding plugin which returned coordinates. The distances between the CRC and the FSA geographical centres were then calculated using Vector/Analysis Tools/Distance Matrix, returning distance values in metres which were converted to km. Additionally, median household income was retrieved from Statistics Canada’s 2016 census profile [21] for every FSA in the dataset. A subset of the sample containing only unique individuals was created to generate income quintiles. As a result, if repeat visits occurred more often by individuals in particular income quintiles, those income quintiles would be overrepresented across visits.

#### Clinical characteristics

For each visit, we determined if the individual had a prior visit to the CRC within 1 year. Suicidal behaviour was entered by the assessing clinician who selected the highest level of none, ideation, planning or attempt/self harm behaviour during that presentation. Diagnostic impression was also entered by the assessing clinician into non-mutually exclusive categories based on their assessment and available collateral information. Categories included depression or anxiety (included adjustment problems, sleep problems, obsessive compulsive and trauma-related problems), psychosis, bipolar spectrum disorder, cognitive impairment (dementia, delirium, intellectual disability, acquired brain injury, autism), and other (primarily attention deficit hyperactivity disorder, eating disorders and other impulse control problems). Presence of substance use was coded as present or absent.

#### Outcome of visit

The occurrence of a psychiatric consultation was coded as yes or no. Dispositions included discharge, hospitalization, or referral to a brief stay crisis stabilization unit (CSU) or the virtual ward.

### Data analysis

In-person and virtual visits were separately binned by week and the weekly proportion of visits delivered virtually was plotted across the study period. The average proportion of visits during each pandemic period was calculated. A one-way analysis of variance (ANOVA) with Tukey’s adjustment method for multiple comparisons was used to examine the differences in the percent of virtual visits across the five different pandemic periods. For the comparison sample analysis, 4,253 visits were retrieved. We used the distance variable to restrict to cases where either virtual or in person care could be expected to be equally available options, and the distance variable would be a reliable indicator. We first restricted the dataset to visits where the individual’s residence was known and within Manitoba (214 visits excluded; 9 confirmed as non-Manitoba addresses and the rest missing for reasons that may include that no address was recorded in the EPR or the individual was homeless). We then restricted to distance < 30 km from the CRC because on exploration of the data, values above this were categorized as extreme outliers. These outliers were suspected to represent cases of people not living in Winnipeg who happened to be in the city at the time of the crisis (eg. residence distance of 800 km). When we examined a map, the 30 km radius was representative of the geography that most CRC users come from. An additional 183 visits were excluded by this criterion. Finally, we excluded 283 visits where no MHA occurred, rather the individual was seen immediately by the psychiatry team due to acuity of the presentation. Twelve (4.2%) of these occurred virtually, with the remainder in person at the CRC. We generated descriptive statistics of the visits overall and stratified by type of visit (in person or virtual). For all visits that received an MHA, a binary logistic regression was done to examine associations between the visit characteristics, sociodemographics and clinical characteristics and the type of visit. Visit outcomes were compared between groups with chi-squared tests of independence.

## Results

**Fig. 1:**
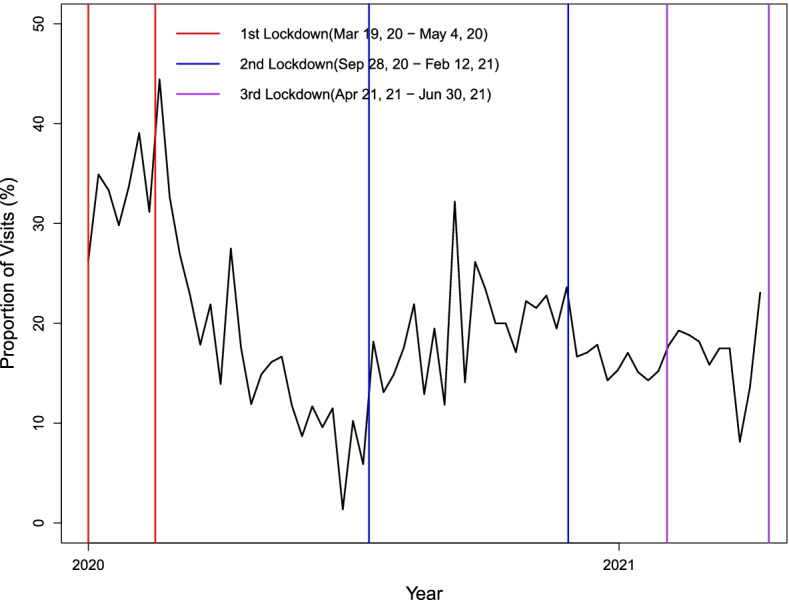
Proportion of visits that occurred virtually from the first lockdown on March 19, 2020 to June 30, 2021

There was a total of 5,357 visits from the onset of the first lockdown to the end of the study period (March 19, 2020 to June 30, 2021), with 993 (18.5%) occurring virtually. The proportion of visits that were virtual varied across the pandemic periods (Fig. 1). The highest uptake of virtual visits occurred during lockdown 1 (average of 32.6% of visits by week) and then a decrease but a sustained level of virtual care throughout the rest of the pandemic (Table 1). There were significant differences in the percent of virtual visits among the five pandemic periods (*F*(4, 62) = 8.56, *p* < 0.001). Multiple comparison procedure with Tukey’s studentized range test indicated that the average percent of virtual visits during the first lockdown was significantly higher than each of the other four time periods, with no significant differences among the other four time periods.

**Table 1:** Total and mean weekly visits and proportion of visits that were virtual by pandemic period

Period	Number of weeks	Total Visits Mean (*SD*)	Virtual Visits Mean (*SD*)	% Virtual (*SD*)
03–19-2020 to 05–04-2020 (1^st^ lockdown)	7	62.9 (10.1)	20.6 (4.4)	32.6 (4.0)
05–05-2020 to 09–27-2020 (After 1^st^ lockdown)	21	82.9 (9.7)	14.2 (8.4)	16.9 (9.8)
09–28-2020 to 02–12-2021 (2^nd^ lockdown)	19	74.9 (8.8)	14.4 (3.4)	19.4 (5.0)
02–13-2021 to 04–20-2021 (After 2^nd^ lockdown)	10	85.0 (8.2)	14.0 (1.4)	16.6 (2.7)
04–21-2021 to 06–30-2021 (3^rd^ lockdown)	10	81.1 (8.2)	13.6 (2.7)	17.0 (3.9)

Lockdowns correspond to period of heightened public health restrictions and stay at home orders

The comparison sample comprised 3,573 total visits; 2,802 (78.4%) were in person and 771 (21.6%) were virtual. There were 2,776 unique individuals in the sample; 78.3% (*n* = 2,195) of in person visits were for unique individuals, and 92.6% (*n* = 714) of virtual visits. Both types of visits occurred for 133 individuals (4.8% of the total individuals). The visit characteristics are presented in Table 2, overall and stratified by visit type. Virtual visits occurred more often on weekdays, during daytime hours, and among female patients. There tended to be lower rates of suicidal behaviour, less substance use, less psychosis and cognitive impairment among the people who were assessed virtually, as well as higher rates of depressive or anxiety problems. The average distance the individual lived from the CRC was farther among virtual visits compared to in person (7.5 vs 6.6 km).

**Table 2:** Baseline characteristics of visits receiving a mental health assessment

Baseline variable	Overall *N* = 3573	Virtual *n* = 771	In-person *n* = 2802
Pandemic Timeline, *n* (%)			
Lockdown 1	340 (9.5)	121 (15.7)	219 (7.8)
In between period	1450 (40.6)	286 (37.1)	1164 (41.5)
Lockdown 2	1228 (34.4)	263 (34.1)	965 (34.4)
After lockdown 2	555 (15.5)	101 (13.1)	454 (16.2)
Time of Day, *n* (%)			
7AM-3PM	1569 (43.9)	384 (49.8)	1185 (42.3)
3PM-11PM	1595 (44.6)	338 (43.8)	1257 (44.9)
11PM-7AM	409 (11.4)	49 (6.4)	360 (12.8)
Day of Week, *n* (%)			
Weekday (Mon-Thurs)	2185 (61.2)	510 (66.1)	1675 (59.8)
Weekend (Fri-Sun)	1388 (38.8)	261 (33.9)	1127 (40.2)
Age, mean (*SD*)	33.9 (13.4)	35.2 (14.0)	33.6 (13.2)
Gender, n (%)			
Male	1555 (43.5)	282 (36.6)	1273 (45.4)
Female	1981 (55.4)	481 (62.4)	1500 (53.5)
Other	30 (0.8)	3 (0.4)	27 (1.0)
Missing	7 (0.2)	5 (0.6)	2 (0.1)
Income Quintile, *n* (%)			
Q1 (lowest)	807 (22.6)	131 (17.0)	676 (24.1)
Q2	804 (22.5)	169 (21.9)	635 (22.7)
Q3	762 (21.3)	178 (23.1)	584 (20.9)
Q4	616 (17.2)	132 (17.1)	484 (17.3)
Q5 (highest)	582 (16.3)	161 (20.9)	421 (15.0)
Missing	2 (0.1)	0 (0.0)	2 (0.1)
Distance in km, mean (*SD*)	6.8 (4.3)	7.5 (4.2)	6.6 (4.2)
Prior visit in last year, *n* (%)	1152 (32.2)	185 (24.0)	967 (34.5)
Substance Use, *n* (%)	1616 (45.2)	249 (32.3)	1367 (48.8)
Level of suicide behaviour, *n* (%)			
None	1608 (45.0)	417 (54.1)	1191 (42.5)
Ideation	1119 (31.3)	216 (28.0)	903 (32.2)
Planning	274 (7.7)	41 (5.2)	233 (8.3)
Self-harm/Attempt	572 (16.0)	97 (12.6)	475 (17.0)
Diagnostic Impression, *n* (%)^a^			
Cognitive Impairment	213 (6.0)	24 (3.1)	189 (6.7)
Personality Problem	888 (24.9)	184 (23.9)	704 (25.1)
Depressive or Anxiety Problem^b^	2592 (72.5)	633 (82.1)	1959 (69.9)
Psychosis	707 (19.8)	73 (9.5)	634 (22.6)
Bipolar Spectrum Disorder	246 (6.9)	49 (6.4)	197 (7.0)
Other^c^	234 (6.5)	41 (5.3)	193 (6.9)

^a^ Categories are not mutually exclusive and coded as present or absent

^b^ Includes adjustment problems, sleep disorders, obsessive compulsive and trauma-related problems

^c^ Includes attention deficit hyperactivity disorder, eating disorders, other impulse control problems

The multiple logistic regression was used to examine how these characteristics associated with the likelihood of a virtual visit. Ten visits missing data in one or more variables were excluded from this analysis. The results are shown in Table 3. In the adjusted model (Table 3), virtual visits were significantly less likely during each pandemic period after the first lockdown, with overnight visits compared to daytime visits (OR 0.48, 95% CI 0.34-0.67, *p* < 0.001), and weekend visits compared to weekday visits (OR 0.75, 95% CI 0.61-0.91, *p* = 0.004). Older age (OR 1.01, 95% CI 1.00–1.01, *p* = 0.025) was associated with receipt of a virtual visit. Compared to females, males were less likely to have a virtual visit (OR 0.76, 95% CI 0.64-0.91, *p* = 0.002), as were those with prior year contact with the CRC vs those without (OR 0.75, 95% CI 0.61-0.91, *p* = 0.004). With respect to clinical characteristics, virtual visits were less likely with all levels of suicidal behaviour compared to no suicidal behaviour, and in the presence of substance use, cognitive impairment, and psychosis. Living further from the CRC was associated with higher odds of receiving a virtual visit (OR 1.04, 95% CI 1.02–1.07, *p* < 0.001).

**Table 3:** Binary logistic regression results for a virtual visit compared to an in person visit (*N* = 3,563)

Baseline variable	OR	95% CI	*p*-value
Pandemic Timeline (reference: Lockdown 1)			
In between period	.37	.28, .49	< .001
Lockdown 2	.39	.30, .52	< .001
After lockdown 2	.35	.26, .49	< .001
Time of Day (reference: 7AM-3PM)			
3PM-11PM	.89	.74, 1.06	.175
11PM-7AM	.48	.34, .67	< .001
Day of Week (reference: weekday, Mon-Thurs)			
Weekend (Fri-Sun)	.75	.61, .91	.004
Age	1.01	1.00, 1.01	.025
Gender (reference: Female)			
Male	.76	.64, .91	.002
Other	.43	.13, 1.45	.175
Income Quintile (reference: Q1, lowest)			
Q2	1.32	1.01, 1.74	.046
Q3	1.20	.91, 1.57	.198
Q4	.94	.70, 1.27	.689
Q5 (highest)	1.29	.96, 1.74	.096
Distance	1.04	1.02, 1.07	.001
Prior visit in last year (reference: no)	.75	.61, .91	.004
Substance Use (reference: no)	.60	.50, .72	< .001
Level of suicidal behaviour (reference: none)			
Ideation	.74	.61, .90	.003
Planning	.55	.38, .79	.001
Self-harm/Attempt	.62	.48, .81	< .001
Cognitive Impairment (reference: absent)	.53	.34, .84	.007
Personality Problem (reference: absent)	1.13	.92, 1.40	.255
Depressive or Anxiety Problem^a^ (reference: absent)	1.26	.98, 1.63	.074
Psychosis (reference: absent)	.41	.30, .56	< .001
Bipolar Spectrum Disorder (reference: absent)	.94	.65, 1.34	.720
Other^b^ (reference: absent)	.82	.57, 1.19	.303

^a^ Includes adjustment problems, sleep disorders, obsessive compulsive and trauma-related problems

^b^ Includes attention deficit hyperactivity disorder, eating disorders, other impulse control problems

Table 4 compares the visit outcomes between in person and virtual visits. There was a significant effect of visit type on disposition (χ^2^(3) = 130.5, *p* < 0.001); compared to virtual visits, in person visits were significantly more likely to have a psychiatric assessment conducted (25.2% vs 5.7%) and result in hospitalization (15.3% vs 2.2%) whereas virtual visits more often resulted in a virtual ward admission (10.8% vs 6.3%).

**Table 4:** Outcomes for all visits receiving a mental health assessment

Outcome	Overall	Virtual	In-person	*p*-value
Psychiatric assessment done, *n* (%)	750 (21.0)	44 (5.7)	706 (25.2)	< .001
Disposition, *n* (%)				< .001
Discharge	2335 (65.4)	590 (76.5)	1745 (62.3)	
CSU^a^	532 (14.9)	81 (10.5)	451 (16.1)	
Hospitalization	445 (12.5)	17 (2.2)	428 (15.3)	
Virtual Ward^b^	261 (7.3)	83 (10.8)	178 (6.3)	

^a^ *CSU* Crisis Stabilization Unit

^b^ Virtual Ward: included a virtual Crisis Stabilization Unit and a virtual psychiatric reassessment and observation unit

## Discussion

In this study we have illustrated the naturalistic uptake of virtual crisis assessments at our standalone 24/7 mental health facility that continued to offer walk-in care during the pandemic. There was a large spike in virtual visits during the first COVID-19 lockdown (> 30% of assessments virtual), followed by a decrease to approximately 15–20% that was sustained out to the end of the third lockdown. We identified that a number of individual and visit-related factors were associated with receipt of a virtual visit, including weekday daytime visits, age, female gender, residence further from the CRC, no prior year contact with the CRC, and the absence of addiction, suicidal behaviour, cognitive impairment, and psychosis. Virtual visits less often resulted in hospitalization, and were more likely to be referred to virtual follow-up care.

The trend in virtual service uptake appears to align with the general populations’ behavior and emotions at the outset of the pandemic. The large spike in virtual care visits during the first lockdown likely represent an avoidance of healthcare facilities (and outings in general), in the context of public health restrictions and anxiety about COVID-19 infection. Several studies documented medical care avoidance during the pandemic, resulting in an initial decline in emergency department visits and hospital admissions [22–24]. Ganson and colleagues [25] found that individuals with depression and anxiety were more likely to avoid seeking non-coronavirus medical care despite needing it most. The availability of virtual mental health assessments that could be initiated directly from a crisis line phone call could mitigate some of this anxiety and serve as an acceptable way to receive needed care. Concurrent with public anxiety, there was a massive push within health systems to deliver virtual services to provide the same scope of care [25]. This resulted in our local staff initially being very vigilant about encouraging virtual assessments, including redirecting individuals with lower acuity mental health needs to call rather than present in person at the CRC, with daily huddles, leadership guidance, and clinical supervision strongly encouraging this practice during the first lockdown.

As the pandemic progressed, the fears pertaining to the exposure of COVID in the general public and among staff began to dissipate [26, 27] as personal protective equipment was secured and required screening and care delivery practices became routine. This change parallels the trend seen in virtual uptake in this study, with decreased virtual visits after the first lockdown and no differences between subsequent time periods regardless of lockdown status. What is noteworthy is the sustained level of virtual visits through the remaining study period. The need to rapidly transform services and the collective anxiety about COVID-19 created sufficient extrinsic motivation to overcome many of the usual barriers to practice change in healthcare [28, 29]. The routine delivery of virtual assessments and system-wide support of virtual care likely contributed to its maintenance as a care delivery mechanism. Although some clients undoubtedly continued to prefer this option out of COVID-19 related fear [25], we also suspect that staff came to see the benefit of the virtual assessment not only in terms of reducing covid risk, but also as a person-centred option for clients. As Breckenridge et al. [28] have posited, this creates a dynamic whereby evidence of effective change becomes evidence to sustain the change. Sustained practice change requires staff appreciate the value of the change, concurrent with the alignment of leadership, policy, capacity building, among other organizational factors [28, 29]. The pandemic has catalyzed these changes but longer-term sustainability will require ongoing attention to these other contributors [5, 30].

The associations with specific individual and visit-related factors highlights particular instances when virtual care is more likely to be offered and/or requested and those profiles that may be most appropriate. Virtual visits were more likely when the mental health acuity was lower. This fits with the criteria used by staff to determine appropriateness to send someone home to receive a virtual assessment, and with the likelihood for certain high-risk presentations to present in person rather than calling in through the crisis line (eg. disorganized or dangerous behaviour resulting in involvement of authorities). There is literature to support that presentations of psychosis and suicide can be assessed remotely by video [31], however, these presentations may have safety factors to consider and in some cases may feature emotional nuances that are more difficult to assess virtually [10]. Although presentations with active suicidality or psychosis were less likely in the virtual group, they still occurred, suggesting that virtual assessment was feasible in some cases. Reduced receipt of virtual visits with certain clinical presentations could reflect individual or family care seeking preferences, severity of presentation requiring a contained environment, technology literacy or staff comfort. Further capacity building may influence staff decision to offer virtual assessment, in addition to a better understanding of patient preferences. Female clients were significantly more likely to receive virtual care which could be explained by increased help-seeking behaviour, particularly in the context of mood or anxiety problems [32], or the increased likelihood that females are balancing other demands that could impede an in person visit such as child care [33].

This study presents a unique situation where, unlike acute care services that converted 100% to virtual [17], the CRC continued to offer in person assessments which allowed the examination of service uptake and factors associated with receipt of virtual care. There are still some limitations to be considered. First and foremost, the pandemic created an unexpected catalyst for the adoption of virtual care. The duration of this study is within the pandemic, so it is still not known what the longer-term uptake of virtual crisis assessments will be. We used the visit as the unit of analysis to be able to examine visit related factors such as time of day and day of week. However, this means that some individuals had multiple visits. Visits may have occurred for different clinical reasons, eg. a visit for substance induced psychosis with another later visit for mood related difficulties while sober. Individuals may have been more likely to repeat certain visit types based on their own help seeking preferences or interactions with other services, although we also had 133 individuals with repeat visits of both types.

There are several data limitations. For example, diagnostic impressions were based on clinician assessment and may be subject to variability in knowledge and experience. Distance and income were derived from postal code on file at the time of data retrieval, and individuals may have moved. Additionally, individuals with missing postal codes were not included as we could not verify if the postal code was not collected or if the individual was homeless. We cannot discern which virtual visits were by telephone or video and we did not have several potentially important variables such as patient preference or availability of support persons at the time of the assessment. Questions about access and preference are important as research shows that people of lower income, lower education level, or immigration status are less likely to have access to computers and high-speed internet [34, 35]. Our EPR does not reliably capture ethnicity so we could not examine how this factor may impact receipt of services. Additionally, concerns about privacy and confidentiality may be more likely to affect lower income individuals including small residences with lack of private space [36]. We did not examine calls to the crisis line in this study, but looking at program data for the 2020–2021 fiscal year and the year prior we noted a small increase in volume of calls (from 10,573 calls in 2019–2020 to 11,780 in 2020–2021). Without additional data analysis, we don’t know if this is a significant increase, but it is definitely possible that certain individuals may have chosen to make first contact by phone due to COVID concerns, resulting in a higher likelihood of a virtual MHA being offered. As a result, the comparison groups are subject to selection bias. Still, this naturalistic study represents important learning for the delivery of virtual crisis care outside of the pandemic.

## Conclusions

As we move forward, there will continue to be increased support and investment for virtual care now that the potential has been widely recognized [5, 30]. We have shown that there is a place for this mode of assessment in the crisis setting, albeit definitely not a replacement for usual in person care. We will need to continue to understand more about how and when virtual care should be offered ensuring equitable access, and to what extent we can accurately conduct crisis assessments. We present early evidence of sustained practice change in our crisis setting supporting that both staff and the public are receptive to this type of care delivery.

## Data Availability

Data generated for the current study are not publicly available for ethical reasons. Data may be made available for researchers who meet the criteria for access to confidential data upon a reasonable request to the corresponding author.

## Abbreviations

COVID-19: Coronavirus disease 2019
CRC: Crisis Response Centre
CSU: Crisis Stabilization Unit
MHA: Mental health assessment
EPR: Electronic patient record
FSA: Forward sortation area
CSU: Crisis Stabilization Unit
ANOVA: Analysis of variance
